# Обзор распространенности и особенностей онкологических заболеваний при сахарном диабете 2 типа

**DOI:** 10.14341/probl13452

**Published:** 2025-05-20

**Authors:** Я. В. Дворяньчиков, С. М. Деунежева, И. А. Яцков, В. А. Белоглазов

**Affiliations:** Национальный медицинский исследовательский центр эндокринологии; Национальный медицинский исследовательский центр эндокринологии; Крымский федеральный университет имени В.И. Вернадского; Крымский федеральный университет имени В.И. Вернадского

**Keywords:** сахарный диабет 2 типа, онкологические заболевания, гиперинсулинемия, гипергликемия

## Abstract

Во всем мире число пациентов с сахарным диабетом увеличилось в четыре раза за последние три десятилетия. Каждому 11-му взрослому человеку в настоящее время выставлен диагноз: «Сахарный диабет», 90% из которых — сахарный диабет 2 типа (СД2). К общепризнанным осложнениям хронической гипергликемии относят микро- и макрососудистые изменения, повреждения периферических и/или автономных нервных волокон. Также учеными давно обсуждается взаимосвязь между увеличением числа определенных онкологических заболеваний и наличием СД2. Исходя из наличия общих факторов риска, таких как возраст, этническая принадлежность, особенности питания и уровень физической активности, проводятся многие эпидемиологические и экспериментальные исследования, которые постепенно вносят свой вклад в понимание взаимосвязи между данными заболеваниями. Принимая во внимание результаты многочисленных исследований, гипергликемия, гиперинсулинемия и хроническое воспаление, которые наблюдаются при СД2, имеют положительную связь с повышенным риском возникновения определенных видов злокачественных образований. В данной статье авторы рассматривают патологические изменения при СД2, потенцирующие развитие онкологических заболеваний и эпидемиологические данные, отражающие корреляцию между СД2 и возникновением злокачественных образований.

## Abstract (en)

Worldwide, the number of patients with diabetes has quadrupled over the past three decades. Every eleventh adult is currently diagnosed with diabetes mellitus, 90% of which are type 2 diabetes mellitus (DM2). Generally recognized complications of chronic hyperglycemia include micro- and macrovascular changes, damage to peripheral and/or autonomous nerve fibers. Scientists have also long discussed the relationship between an increase in the number of certain cancers and the presence of DM2. Based on the presence of common risk factors such as age, ethnicity, dietary habits and physical activity, many epidemiological and experimental studies are being conducted, which gradually contribute to understanding the relationship between these diseases. Taking into account the results of numerous studies, hyperglycemia, hyperinsulinemia and chronic inflammation, which are observed in DM 2, have a positive association with an increased risk of certain types of malignancies. In this article, the authors consider pathological changes in DM2 that potentiate the development of oncological diseases and epidemiological data reflecting the correlation between DM2 and the occurrence of malignant tumors.

## ВВЕДЕНИЕ

На протяжении десятилетий в ряде когортных исследований сообщалось об увеличении частоты встречаемости гепатоцеллюлярной карциномы, рака поджелудочной железы, молочной железы, тела матки и колоректального рака — при наличии у пациента сахарного диабета 2 типа (СД2). В то же время для некоторых злокачественных новообразований данного риска выявлено не было (таких, как рак почки, лейкемия и рак пищевода) [1].

Взаимосвязь между аденокарциномой предстательной железы и диабетом уникальна, поскольку это единственный вид рака, при котором диабет оказывает защитное действие. По данным метаанализа 45 обсервационных исследований, было выявлено, что у мужчин с СД2 риск развития рака предстательной железы на 14% ниже, чем у мужчин без диабета, однако у пациентов с СД2 смертность от него выше [3]. Снижение риска может быть объяснено как наличием определенных аллелей гена HNF1B (rs757210), так и статистически более низким уровнем циркулирующих андрогенов. Также имеются также данные о том, что мужчины с диабетом имеют более низкие уровни циркулирующего простатспецифического антигена (ПСА), онкомаркера, который используется для скрининга рака предстательной железы, что может привести к более низкой частоте его выявления [46]. Однако эти данные не коррелируют с полученными результатами крупных метаанализов на азиатской популяции, где было выявлено увеличение частоты встречаемости аденокарциномы предстательной железы у пациентов с СД2 [2].

На фоне многочисленных исследований патофизиологии СД2 было выявлено несколько факторов, способствующих повышенному риску развития онкологических заболеваний. К ним относятся гипергликемия, гиперинсулинемия, повышенная биоактивность инсулиноподобного фактора роста 1, окислительный стресс, нарушение регуляции половых гормонов и хроническое воспаление [6]. Злокачественная трансформация обычно включает процесс инициации (нарушение генетической стабильности), за которым следует промоция, которая включает в себя селективную клональную экспансию инициированных клеток [53], и прогрессия, в ходе которой стимулируется рост и развитие опухолевых клеток. Хронические эффекты эндогенной или экзогенной гиперинсулинемии могут также способствовать злокачественной трансформации через прямые или косвенные механизмы [7].

## ПРЯМЫЕ И КОСВЕННЫЕ ЭФФЕКТЫ ИНСУЛИНА НА РОСТ ОПУХОЛИ

Инсулин и инсулиноподобные факторы роста 1 и 2 (IGF-1 и IGF-2) играют ключевую аутокринную, паракринную и эндокринную роль в стимулировании пролиферации и дифференцировки клеток. IGF проявляют свое действие, связываясь со специфическими гликопротеиновыми мембранными рецепторами, а именно с рецептором IGF-1 (IGF-1R), инсулиновыми рецепторами A и B (IR-A и IR-B) и гибридными рецепторами (IGF-1R/IR-A и IGF-1R/IR-B) [67]. Более того, среди рецепторов IGF наблюдается заметная гомология, что подразумевает структурное сходство и возможность передачи сигналов перекрестных помех [56]. IR экспрессируется не только в поджелудочной железе, жировой ткани, мышечной ткани и печени, которые непосредственно участвуют в синтезе гормонов, метаболизме глюкозы и свободных жирных кислот [54], но и во многих других тканях. Хотя IGF-IR традиционно считался медиатором митогенной передачи сигналов, а IR — активатором метаболической передачи сигналов, фосфорилирование IR фактически активирует как метаболические, так и митогенные сигнальные пути [8]. Кроме того, существует два варианта сплайсинга IR-A и IR-B. В данном процессе экзоны, кодирующие области матричной рибонуклеиновой кислоты, сохраняются, а некодирующие области мРНК — интроны вырезаются и удаляются [55]. IR-A не имеет экзона 11 и в основном экспрессируется в эмбриональных и опухолевых тканях, в то время как IR-B преимущественно экспрессируется в печени, скелетных мышцах, жировой ткани и почках. Экспрессия некоторых факторов сплайсинга определяет соотношение IR-A и IR-B в клетке [9].

Недавние исследования показали, что активация рецептора эпидермального фактора роста (EGF-R)/митоген-активируемой протеинкиназой (MAPK) увеличивает соотношение IR-A/IR-B в клеточных линиях гепатоцеллюлярной карциномы за счет повышения экспрессии определенных факторов сплайсинга в опухолевых клетках, а не в регенерирующих клетках нормальной печени [10]. Изменения в экспрессии факторов сплайсинга, таких как SRSF3, приводят к увеличению экспрессии IR-A и способствуют росту гепатоцеллюлярной карциномы. Воспаление в тканевом микроокружении также может усиливаться гиперинсулинемией, что приводит к локальной продукции провоспалительных цитокинов и активации сигнального пути Jak-Stat в опухоли [11].

## ПРЯМЫЕ И КОСВЕННЫЕ ВЛИЯНИЯ ГИПЕРГЛИКЕМИИ

Повышенная концентрация глюкозы способствует синтезу ДНК опухолевых клеток, поскольку метаболизм злокачественных клеток зависит от пентозофосфатного пути. Феномен, называемый «эффектом Варбурга», частично объясняет, почему гипергликемия способствует онкогенезу.

В норме клетки дифференцируются за счет митохондриального окислительного фосфорилирования, чтобы обеспечить энергией клеточные процессы, в то время как злокачественные клетки как правило используют менее эффективный гликолитический путь для пролиферации [2], что способствует повышенному поглощению глюкозы с целью генерирования достаточного количества энергии и сохранению высокой пролиферативной активности. Кроме того, Хан и соавт. сообщили, что гипергликемия стимулирует пролиферацию клеток рака поджелудочной железы (линий BxPC-3 и Panc-1) через индукцию экспрессии эпителиального фактора роста (EGF) и трансактивацию рецептора EGF [12]. Из-за нарушения окислительного баланса накапливаются конечные продукты гликирования, что приводит к высвобождению свободных радикалов, цитокинов и факторов роста [13]. Данный каскад биохимических реакций приводит к повреждению внеклеточного матрикса и увеличению проницаемости базальной мембраны, тем самым способствуя экспансии злокачественного образования.

Было также показано, что при СД повышается уровень матриксной металлопротеиназы 9 типа, что способствует локальному распространению опухолевых клеток и образованию метастазов [14]. Конечные продукты гликирования часто взаимодействуют со своим специфическим рецептором (RAGE), активируют транскрипционный ядерный фактор kB и генерируют активные формы кислорода в клетках, тем самым ускоряя окислительный стресс, который приводит к усилению провоспалительной сигнализации [12].

## ГЕПАТОЦЕЛЛЮЛЯРНАЯ КАРЦИНОМА

Рак печени остается глобальной проблемой здравоохранения: по оценкам, к 2025 г. заболеваемость превысит 1 миллион случаев. Гепатоцеллюлярная карцинома (ГЦК) является наиболее распространенной формой рака печени и составляет около 90% всех случаев. Заражение вирусами гепатита В и С является основным фактором риска развития ГЦК, хотя неалкогольный стеатогепатит (НАСГ), связанный с метаболическим синдромом или СД, становится все более частым фактором риска [28].

Взаимосвязь между СД и повышенным риском развития ГЦК была впервые представлена Лоусоном в 1986 г. и впоследствии поддержана другими авторами [18–21]. Было установлено, что ГЦК обычно возникает на фоне цирроза печени, однако недавние исследования показали, что в настоящее время ГЦК чаще наблюдается у пациентов с ожирением и резистентностью к инсулину, СД2 и неалкогольной жировой болезнью печени (НАЖБП), чем при циррозе печени [23].

В исследовании Ajmera V и соавт. выявлен высокий риск прогрессирующего фиброза/цирроза печени у взрослых в возрасте ≥50 лет с СД2 [24]. Корейское исследование с использованием когорты Национальной службы медицинского страхования (NHIS-HEALS) показало, что СД2 был связан с более высоким риском ГЦК (коэффициент риска — 1,82) [16], при этом относительный риск значительно выше у мужчин, чем у женщин [26].

Известно, что хронические заболевания печени повышают риск развития СД2. Таким образом, некоторая часть связи между СД2 и ГЦК в общей популяции может быть обусловлена как увеличением риска возникновения ГЦК при СД2, так и при ГЦК возникновением СД2 [22].

Систематический обзор El-Serag и соавт. показал, что СД2 был связан примерно с 2,5-кратным увеличением риска ГЦК. Кроме того, оценка риска из 13 исследований типа «случай-контроль» показала 2,5-кратное увеличение вероятности манифестации диабета у пациентов с ГЦК по сравнению с контрольной группой без диабета [25].

Число случаев ГЦК, связанных с НАСГ, увеличивается, а число случаев, связанных с вирусным гепатитом В или С, снижается благодаря широкому применению всеобщей вакцинации против вируса гепатита В и внедрению эффективных методов лечения вирусного гепатита С [27]. Указанные закономерности позволяют считать, что борьба с ожирением и диабетом является чрезвычайно важными для первичной профилактики НАЖБП и ГЦК.

## РАК ПОДЖЕЛУДОЧНОЙ ЖЕЛЕЗЫ

Рак поджелудочной железы (РПЖ) является ведущей причиной онкологической смертности во всем мире, и его глобальное бремя более чем удвоилось за последние 25 лет [36]. По данным обсервационных исследований, выявлена положительная взаимосвязь между наличием нарушения углеводного обмена и развитием РПЖ, однако и непосредственно сам РПЖ может приводить к нарушению углеводного обмена путем прямого разрушения бета-клеток [15].

По данным Американской диабетической ассоциации, СД2 и диабет типа 3с заслуживают особого внимания в отношении риска развития РПЖ [29], так как являются факторами риска развития аденокарциномы протоков поджелудочной железы. Эти данные подтверждаются в популяционном Тайванском когортном исследовании, в котором риск аденокарциномы протоков поджелудочной железы был существенно повышен у пациентов с сопутствующим СД и хроническим панкреатитом (коэффициент риска — 33,5) [37].

В обзорной статье Jie Cai и соавт. было отмечено, что, по данным ряда метаанализов, продемонстрировано увеличение риска развития РПЖ в 2 раза у пациентов с СД2 (ОР 1,94, 95% ДИ 1,66–2,27). Также было выявлено, что каждое повышение уровня глюкозы в крови натощак на 0,56 ммоль/л было связано с повышением риска развития аденокарциномы поджелудочной железы на 14% [31].

При этом относительный риск развития рака поджелудочной железы отрицательно коррелировал с продолжительностью диабета. В работе Ayush Sharma и соавт. также выявлена обратная зависимость риска РПЖ от продолжительности СД, повышенная частота РПЖ сохранялась только в первые 5 лет после постановки диагноза «СД». Это подчеркивает важность выделения впервые выявленного СД (определяемого как СД длительностью <3 лет) от длительного существующего СД (определяемого как СД продолжительностью >3 лет) при оценке риска РПЖ [35], что согласуется с данными Furong Wang и соавт. о снижении риска развития РПЖ при длительном стаже диабета [34]. Также имеются данные, что впервые выявленный СД, сопровождающийся потерей веса, был связан со значительным увеличением риска РПЖ, и может представлять собой группу высокого риска, над которой необходим более серьезный контроль [33].

Также стоит упомянуть об увеличении встречаемости РПЖ у пациентов с нарушением толерантности к глюкозе (НТГ) и нарушении гликемии натощак (НГТ). В корейском многоцентровом исследовании, включавшем более 8 млн женщин старше 40 лет, наблюдалось увеличение риска развития РПЖ в 1,2 раза у пациентов с НТГ и НГТ по сравнению с контрольной группой [15].

Принимая во внимание повышенную встречаемость РПЖ у пациентов с СД2, диабетом типа 3с, а также крайне неблагоприятный прогноз РПЖ, врачам-клиницистам необходимо иметь крайнюю настороженность при выявлении пациентов с впервые выявленным СД в сочетании со снижением веса, абдоминальным болевым синдромом, а также при сочетании СД и хронического панкреатита.

## КОЛОРЕКТАЛЬНЫЙ РАК

Во всем мире колоректальный рак (КРР) является третьим наиболее распространенным видом злокачественного новообразования с точки зрения заболеваемости (10,2%), а также вторым по уровню общей смертности (9,0%). По прогнозам, к 2035 г. число пациентов с КРР во всем мире достигнет 2,5 млн [38]. Существование молекулярных связей, которые способствуют развитию КРР в популяции СД2, подтверждается существенным количеством эпидемиологических данных [40].

Особого внимания заслуживают два репрезентативных крупномасштабных проспективных когортных исследования. Первое исследование, проведенное China Kadoorie Biobank, в которое вошли более полумиллиона людей среднего и пожилого возраста из десяти различных городских и сельских районов Китая. После 10-летнего наблюдения у пациентов с СД2 был выявлен повышенный риск КРР, ОР составило 1,18, 95% ДИ: 1,04–1,39 [57]. Второе исследование встречаемости КРР у граждан США с наличием СД задокументировало 3000 новых случаев в течение 32 лет наблюдения. Среди мужчин с СД2 ОР составило 1Ю42, 95% ДИ: 1,12–1,81, а среди женщин с СД2 ОР составило 1,17, 95% ДИ: 0,98–1,39 [39].

В дополнение к увеличению риска КРР наличие СД2 является одним из факторов, который ухудшает ответ на химиотерапию первой линии (фторпиримидины, оксалиплатин и/или иринотекан). Также было выявлено, что диабет увеличивает риск рецидива КРР, а также отдаленное метастатическое поражение [41]. Прогностическая роль СД была изучена у 203 пациентов с метастатическим КРР (по шкале общего состояния онкологического больного ECOG 0–1) с ожидаемой продолжительностью жизни >3 месяцев и возрастом <80 лет. После наблюдения в течение 28 месяцев медиана выживаемости составила 9,6 и 17,3 месяца для пациентов с СД2 и без СД2 соответственно (ОР: 2,01; ДИ: 1,11–3,64). При многофакторном анализе с поправкой на возраст (<65 лет против ≥65 лет), пол (мужской или женский), сторону первичной опухоли (левая или правая), метастатическое поражение (один сайт против нескольких сайтов) и ответ на химиотерапию первой линии (контроль заболевания против отсутствия контроля заболевания) данные сохраняли независимую прогностическую ценность [58].

## РАК МОЛОЧНОЙ ЖЕЛЕЗЫ

Рак молочной железы (РМЖ) является наиболее часто встречающимся злокачественным новообразованием у женщин. Распространенность РМЖ у женщин с СД2 превосходит таковую в общей популяции на 15–20%. Более того, уровень общей смертности у женщин с СД2 на 30–60% выше, чем у женщин без диабета, учитывая поправку на стадию распространенности опухолевого процесса [59]. Также было обнаружено, что СД2 увеличивает риск развития трижды негативного рака молочной железы, который отмечается наихудшим прогнозом из-за отсутствия таргетной терапии и повышенного риска метастазирования [44].

Необходимо также отметить, что избыточная масса тела и ожирение приводят к уменьшению частоты выявления новообразований и увеличенных лимфатических узлов методом пальпации, а из-за увеличения плотности ткани молочной железы, наличия кальцификатов данные рентгенологической картины могут интерпретироваться двояко, что может приводить к ложноотрицательным и ложноположительным результатам [47].

В канадском ретроспективном когортном исследовании у женщин с впервые диагностированным РМЖ и наличием СД2 в анамнезе чаще диагностировались поздние стадии распространения РМЖ (II, III или IV), опухоли более 2 см и метастазы в лимфатические узлы, чем у женщин без диабета [45], что коррелирует с данными раннее проведенных исследований.

В связи с многочисленными работами об увеличении риска РМЖ у женщин с СД2 логично было бы предположить оценку влияния гестационного сахарного диабета (ГСД) на риск развития РМЖ. Так как сам ГСД характеризуется гиперинсулинемией и повышением уровня IGF-1, а также приводит к увеличению риска возникновения СД2, который является независимым фактором риска развития РМЖ, были проведены крупные исследования, однако полученные данные оказались дискордантными, что оставляет вопрос влияния ГСД на РМЖ открытым [60].

## РАК ТЕЛА МАТКИ

Рак тела матки (РТМ) является самой частой злокачественной опухолью женских половых органов в развитых странах мира, частота встречаемости которой неуклонно растет. В 2020 г. во всем мире РТМ был диагностирован у 417 367 женщин, с повышенной частотой встречаемости в Северной Америке и Западной Европе [48]. Согласно объединенному анализу эпидемиологических исследований 1971–2014 гг., проведенному в 2016 г., смертность, связанная с РТМ, увеличивалась в среднем на 1,9% в год [49].

Независимым фактором возникновения РТМ является СД2, наличие которого увеличивает риск возникновения РТМ в 2 раза, независимо от наличия ожирения. Подсчитано, что практически 40% всех случаев РТМ связано с наличием диабета и ожирения [51].

В крупном метаанализе, включавшем 31 исследование с суммарным количеством пациентов с РТМ в 55 475 человек, было выявлено, что специфическая смертность у пациентов с РТМ и СД2 увеличена на 15%, а риск общей смертности, а также прогрессирования или рецидива заболевания был на 42% и 23% выше, чем у пациентов без СД2 [61].

Протеомный анализ белка тканей эндометрия у пациенток с СД2 выявил снижение уровня белка WWC3, что может указывать на агрессивный характер [50]. Белки семейства WWC представляют собой цитозольные каркасные белки, о снижении экспрессии которых сообщалось при исследовании немелкоклеточного рака легких, что было связано со снижением дифференцировки клеток, метастазированием, плохим прогнозом, а также низкой выживаемостью [62].

В работе Firouzeh Heidari [52] и соавт. было выявлено повышение уровня белка теплового шока HSP70 у пациентов с СД2 по сравнению с контрольными группами. Принимая во внимание данные обсервационных исследований, сверхэкспрессия HSP70 в опухолевых клетках способствует росту опухоли и устойчивости к терапии за счет активации антиапоптотических и цитопротекторных способностей, а фенотип mHSP70 был описан у высокоагрессивных опухолей, однако эти данные требуют проверки на больших выборках с проспективной оценкой полученных результатов.

## ИНСУЛИНОТЕРАПИЯ И РИСК РАЗВИТИЯ ОНКОЛОГИЧЕСКИХ ЗАБОЛЕВАНИЙ

По данным лабораторных исследований было выявлено увеличение митогенной активности аналогов инсулина, в частности инсулина гларгин, которое может быть объяснено повышенным сродством к IGF-1. В целом, результаты клеточных исследований указывают на повышение уровня роста, дифференцировки и выживаемости клеток, а не на канцерогенный эффект [63]. Хотя в 2009 г. появился ряд обсервационных исследований, которые связали использование инсулина гларгин с увеличением риска развития онкологических заболеваний среди женщин с СД2 [64]. При анализе этих работ был выявлен ряд недостатков: результаты основывались на кратковременном наблюдении, не включались данные компенсации/декомпенсации СД, дозы инсулина и длительности терапии.

В когортном исследовании CARING, включавшем наблюдение в пяти странах с общим количеством 327 112 пациентов, не было различий в риске развития десяти видов злокачественных новообразований при использовании инсулина гларгин или инсулина детемир по сравнению с человеческим инсулином [65].

В исследовании на мышах, склонных к развитию РМЖ, было выявлено, что инсулин гларгин демонстрирует более выраженный пролиферативный эффект в клетках MCF-7 (эпителиоподобной клеточной линии, полученной из инвазивной аденокарциномы протоков молочной железы человека), которые экспрессируют рецепторы IR и IGF-1R. Однако при сравнении экспрессии рецепторов IR и IGF-1R в MNU-индуцированной (N-Нитрозо-N-метилмочевиной) опухоли молочной железы и клетках MCF-7 было обнаружено, что экспрессия IGF-1R была значительно выше в клетках MCF-7, тогда как экспрессия IR была аналогичной [66]. Таким образом, возможно, канцерогенный эффект инсулина гларгина ограничен при наличии высокой экспрессии IGF-1R. При этом необходимы дальнейшие исследования для более детального понимания данного механизма, а также оценка подтипов IR.

## ЗАКЛЮЧЕНИЕ

Учитывая стремительное увеличение числа пациентов с СД2, закономерно и повышение частоты встречаемости онкологических заболеваний. Принимая во внимание повышение как общей, так и специфической смертности, повышение частоты рецидивов, необходимо стратифицировать пациентов с СД2 в отдельную группу для более пристального контроля. Внедрение скрининговых методов будет способствовать раннему выявлению злокачественных образований и улучшать прогноз пациентов и качество жизни. Что же касается инсулинотерапии, то ее роль в канцерогенезе еще предстоит подробно изучить.

## ДОПОЛНИТЕЛЬНАЯ ИНФОРМАЦИЯ

Источники финансирования. Работа выполнена по инициативе авторов без привлечения финансирования.

Конфликт интересов. Авторы заявляют об отсутствии конфликта интересов.

Участие авторов. Все авторы одобрили финальную версию статьи перед публикацией, выразили согласие нести ответственность за все аспекты работы, подразумевающую надлежащее изучение и решение вопросов, связанных с точностью или добросовестностью любой части работы.
